# STAT2 Mediates Innate Immunity to Dengue Virus in the Absence of STAT1 via the Type I Interferon Receptor

**DOI:** 10.1371/journal.ppat.1001297

**Published:** 2011-02-17

**Authors:** Stuart T. Perry, Michael D. Buck, Steven M. Lada, Christian Schindler, Sujan Shresta

**Affiliations:** 1 Division of Vaccine Discovery, La Jolla Institute for Allergy and Immunology, La Jolla, California, United States of America; 2 Department of Microbiology and Immunology, Columbia University, New York, New York, United States of America; Washington University School of Medicine, United States of America

## Abstract

Dengue virus (DENV) is a mosquito-borne flavivirus, and symptoms of infection range from asymptomatic to the severe dengue hemorrhagic fever/dengue shock syndrome (DHF/DSS). High viral loads correlate with disease severity, and both type I & II interferons (IFNs) are crucial for controlling viral replication. We have previously reported that signal transducer and activator of transcription (STAT) 1-deficient mice are resistant to DENV-induced disease, but little is known about this STAT1-independent mechanism of protection. To determine the molecular basis of the STAT1-independent pathway, mice lacking STAT1, STAT2, or both STAT1 and STAT2 were infected with a virulent mouse-adapted strain of DENV2. In the first 72 hours of infection, the single-deficient mice lacking STAT1 or STAT2 possessed 50–100 fold higher levels of viral RNA than wild type mice in the serum, spleen, and other visceral tissues, but remained resistant to DENV-induced death. In contrast, the double-deficient mice exhibited the early death phenotype previously observed in type I and II IFN receptor knockout mice (AG129), indicating that STAT2 is the mediator of the STAT1-independent host defense mechanism. Further studies demonstrated that this STAT2-dependent STAT1-independent mechanism requires the type I IFN receptor, and contributes to the autocrine amplification of type I IFN expression. Examination of gene expression in the spleen and bone marrow-derived macrophages following DENV infection revealed STAT2-dependent pathways can induce the transcription of a subset of interferon stimulated genes even in the absence of STAT1. Collectively, these results help elucidate the nature of the poorly understood STAT1-independent host defense mechanism against viruses by identifying a functional type I IFN/STAT2 signaling pathway following DENV infection *in vivo*.

## Author Summary

Dengue virus (DENV) is a mosquito-borne pathogen present in the tropical and sub-tropical regions of the world, and an estimated 2.5 billion people are at risk of infection. Interferon (IFN) mediated innate responses in the host are critical for limiting viral spread following DENV infection. We have previously demonstrated that mice lacking STAT1, a key mediator of both type I and II IFN responses, are not susceptible to DENV-mediated disease. In this study, we sought to determine the mechanism responsible for protection against DENV disease in the absence of STAT1. Using knockout mice, we identify STAT2 as the protein that mediates type I IFN signaling during DENV infection in the absence of STAT1. The resulting antiviral response includes amplification of type I IFN and the expression of interferon stimulated genes. These data suggest DENV infection is especially sensitive to STAT2-mediated antiviral responses *in vivo*, and provide novel insights towards how IFNs protect against viral infections.

## Introduction

Interferons (IFNs) play a key role in the defense against viruses and intracellular bacteria [1]–[3]. The type I (α/β) IFN receptor is a heterodimer consisting of the IFN-α/β R1 and R2 chains, binding all of the closely related IFN-α subtypes, as well as IFN-β. The type II (γ) IFN receptor is a distinct heterodimer, consisting of the IFN-γ R1 and R2 subunits, and it binds IFN-γ. Both IFN receptors signal through the Janus kinase-signal transducer and activator of transcription (JAK-STAT) pathway [4], [5] in which JAKs phosphorylate STATs, which then translocate to the nucleus to induce the expression of IFN-stimulated genes (ISGs) [6]. Type I IFN signaling activates STAT1 and STAT2 to heterodimerize and associate with interferon regulatory factor-9 (IRF9) to form IFN-stimulated gene factor-3 (ISGF3), which specifically binds IFN-stimulated response elements (ISREs) within antiviral gene promoters. Type II IFN signaling activates STAT1, which homodimerizes and binds to DNA at γ-activated sequence (GAS) elements.

Studies using mice with targeted disruption of individual IFN receptor and STAT genes have provided valuable insights into how type I and II IFNs function during infection by various pathogens. Mice lacking type I IFN receptor are highly susceptible to infection by viruses such as vesicular stomatitis virus (VSV), lymphocytic choriomeningitis virus (LCMV), vaccinia virus (VV), Semliki Forest virus (SFV), and Theiler's murine encephalomyelitis virus (TMEV) [3]. In contrast, mice deficient in type II IFN receptor are unable to control infection by intracellular bacteria such as *Listeria monocytogenes* and *Mycobacterium tuberculosis*, but are able to mount an effective response against VSV and SFV [3]. Similarly, STAT2-deficient mice exhibit reduced responsiveness to type I IFN and are more susceptible to viral infection [7], whereas STAT1-deficient mice are defective in their response to both type I and II IFNs and are highly sensitive to both viral and intracellular bacterial infections [8], [9]. These studies highlight the critical roles STAT proteins play in protection against viral infection, and why STAT proteins are targeted by viruses to evade antiviral responses.

The *flaviviridae* family includes dengue (DENV), yellow fever (YF), West Nile (WNV), and Japanese encephalitis (JEV) viruses, and represent a group of pathogens that cause significant morbidity and mortality in humans. Several studies have demonstrated that flaviviruses interfere with antiviral responses by targeting STAT1- and STAT2-mediated signaling. Infection with either WNV or DENV inhibits IFN-mediated STAT1 activation *in vitro*, including primary human DC cultures [10]–[14]. JEV and Kunjin virus (a subtype of WNV) infection blocks IFN-α-induced phosphorylation of both STAT1 and STAT2 in multiple cell lines [10], [11], [15]. The antagonists of the antiviral response are primarily the nonstructural proteins of the flaviviruses, which are expressed during infection and required for replication. The expression of NS4B from WNV, YF, or DENV is sufficient for inhibition of IFN-β-induced STAT1 activation in Vero cells, and DENV NS2A and NS4A can contribute to this function [13], [14]. Similarly, the individual expression of multiple Kunjin virus nonstructural proteins can inhibit IFN-α-mediated STAT2 activation in Vero cells [11]. In addition, DENV NS5 binds and targets STAT2 for proteasome-mediated degradation [16]–[18], leading to the loss of STAT2 expression observed during DENV infection *in vitro* [19].

Although STAT1 is a critical component of both type I and II IFN-mediated antiviral responses, STAT1-deficient mice are more resistant to infection with Sindbis virus or murine cytomegalovirus (MCMV) than type I and II IFN receptor-deficient (AG129) mice [20], suggesting that IFN receptors also signal through STAT1-independent mechanisms [21]–[23]. Previously, we have demonstrated that STAT1-deficient 129/Sv mice are resistant to DENV–induced disease, whereas AG129 mice succumb to DENV infection, indicating both STAT1-dependent and STAT1-independent pathways protect against primary DENV infection [24]. However, little is known about the mechanism(s) responsible for STAT1-independent protection against viral infections. In this study we used mice lacking STAT1, STAT2, both STAT1 and STAT2, or both STAT1 and the type I IFN receptor to identify STAT2 as a required mediator of the STAT1-independent antiviral response to DENV infection via the type I IFN pathway. In STAT1-deficient mice, STAT2-dependent mechanisms are responsible for the induction of type I IFN and the expression of a subset of ISGs. These data demonstrate the importance of IFN-dependent, STAT1-independent protection against viral infections *in vivo*, and define key components of protective immunity against DENV.

## Results

### STAT2 mediates STAT1-independent protection against DENV infection in mice

**Figure 1:**
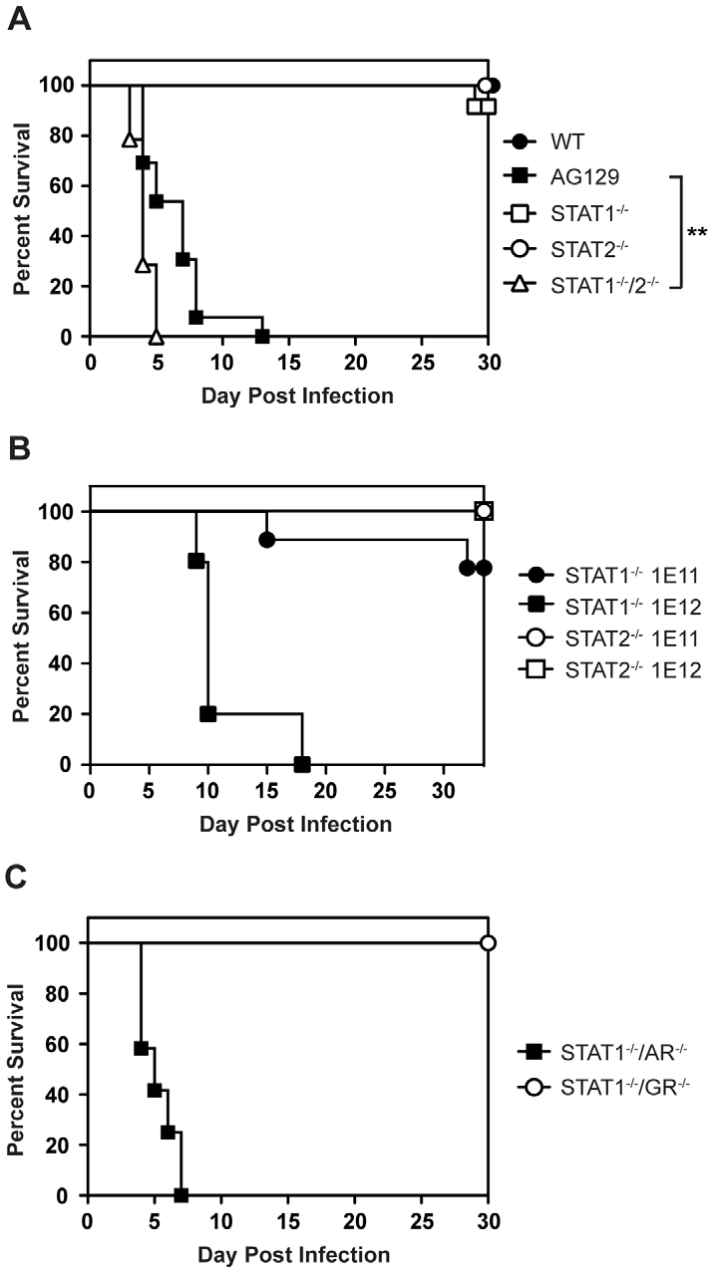
Survival of mice following infection with DENV. Survival was monitored for at least 30 days in mice infected i.v. with DENV2 strain S221. (A) Mice were infected with 10^10^ GE (WT n = 8; STAT1^−/−^ n = 12; STAT2^−/−^ n = 17; STAT1^−/−^/2^−/−^ n = 14; AG129 n = 13). Differences in survival were statistically different (**, p<0.005) between STAT1^−/−^/2^−/−^ and AG129 mice. (B) Mice were infected with 10^11^ GE (n = 9) or 10^12^ GE of DENV (n = 5). (C) Mice were infected with 10^10^ GE (STAT1^−/−^/AR^−/−^ n = 12; STAT1^−/−^/GR^−/−^ n = 8).

Mice lacking type I and type II IFN receptors (AG129), but not their wild type or single-deficient counterparts, are highly susceptible to DENV infection and display an early death phenotype when infected with the DENV serotype 2 (DENV2) strain S221 [25]–[27]. In agreement with published studies showing that type I and type II IFNs restrict DENV infection in mice [24], [26], 12 of 13 AG129 mice died by day 8 following infection with 10^10^ genomic equivalents (GE) (≈2×10^5^ PFU) of the DENV2 strain S221 [28], [29] **(** **Figure 1A** **)**. Although the STAT1 protein is a critical component of both type I and II IFN receptor pathways, mice lacking STAT1 are able to clear DENV infection [24]. Because STAT2-dependent activity has been previously demonstrated downstream of both type I and type II IFN receptors in the absence of STAT1 [30], [31], we hypothesized STAT2 was involved in an IFN-dependent STAT1-independent mechanism of protection against DENV. To assess the contribution of STAT2 to the STAT1-independent control of DENV infection *in vivo*, STAT1^−/−^/2^−/−^ double knockout mice were generated by intercrossing single-deficient animals, and also infected with 10^10^ GE S221. At this dose of virus, 90–100% of wild type and congenic single-deficient mice lacking either STAT1 or STAT2 survived DENV infection. However, all mice with a combined deficiency of STAT1 and STAT2 displayed the early death phenotype observed in AG129 mice and succumbed to DENV infection within 4–6 days, demonstrating the importance of STAT2 to the STAT1-independent mechanism of protection against DENV infection *in vivo* **(** **Figure 1A** **)**. The median survival of STAT1^−/−^/2^−/−^ mice was significantly lower than AG129 at this virus dose (p = 0.0013; **), indicating the absence of both STAT1 and STAT2 renders these mice more susceptible to DENV-mediated disease than the combined loss of type I and II IFN receptors.

To test the limits of this STAT1-independent protection against DENV, the single-deficient mice were infected with 10-fold (10^11^ GE) and 100-fold higher (10^12^ GE) doses of S221 and monitored for survival. Survival proportions of STAT1^−/−^ mice challenged with 10^11^ GE DENV remained the same as those infected with 10^10^ GE DENV, whereas 4 of 5 STAT1^−/−^ animals receiving the highest dose of virus (10^12^ GE) were moribund by day 10 **(** **Figure 1B** **)**. In contrast to STAT1^−/−^ mice, all STAT2^−/−^ animals infected with the equivalent doses of DENV survived beyond 30 days, indicating that although the STAT1-independent antiviral response is effective against DENV *in vivo*, it is less robust than the antiviral response in the absence of STAT2.

### Type I IFN receptor mediates STAT1-independent, STAT2-dependent signaling in response to DENV infection in mice

STAT2 function has been primarily linked to the type I IFN receptor, where it is well-documented to form the ISGF3 complex with IRF9 and STAT1. However, recent data have indicated that STAT2 functions not only downstream of type I IFN, but also type II IFN [30], [32], and type III (λ) IFN signaling [33], implicating type I, type II, and type III IFN receptors as potential candidates for mediating STAT1-independent, STAT2-dependent signaling during DENV infection. Because we observe the early death phenotype in AG129 mice, which retain the type III IFN receptor, we reasoned STAT2 activation was primarily occurring downstream of either the type I or type II IFN receptor. Therefore, to determine which IFN receptors are involved in the STAT1-independent pathway, double-deficient mice lacking STAT1 and either the type I IFN receptor (IFNAR) or type II IFN receptor (IFNGR) were generated by intercrossing the single-deficient mouse strains. Following intravenous infection with 10^10^ GE of S221, 100% of STAT1^−/−^/AR^−/−^ mice developed early lethal disease, whereas none of the STAT1^−/−^/GR^−/−^ mice succumbed to infection **(** **Figure 1C** **)**, demonstrating that the survival phenotype of STAT1^−/−^/AR^−/−^ mice recapitulates that of STAT1^−/−^/2^−/−^ mice. This result indicates that the STAT1-independent, STAT2-dependent mechanism of protection against DENV infection in mice is mediated by the type I IFN receptor.

### The combined loss of STAT1 and STAT2 results in high viral burden in tissues

**Figure 2:**
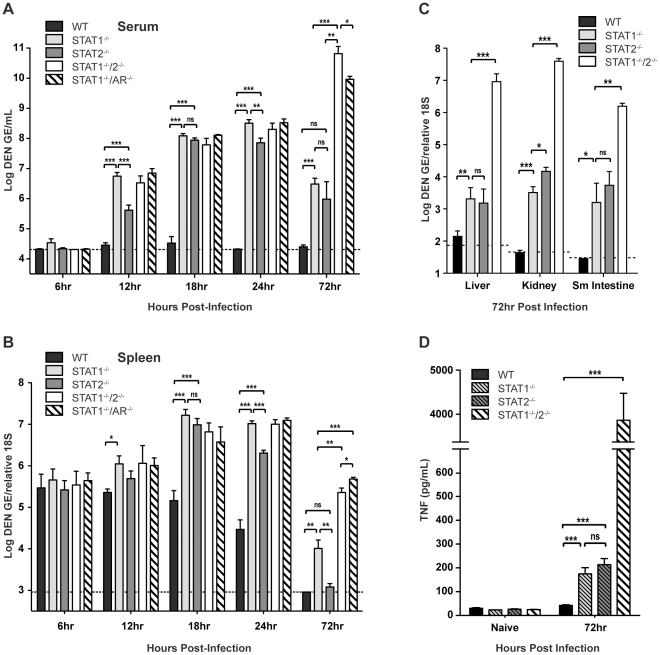
Viral RNA levels in DENV-infected mice. Mice were infected i.v. with 10^10^ GE of S221. (A–C) Quantification of DENV RNA in the (A) serum and (B) spleen at 6, 12, 18, 24 and 72 hours post-infection, and (C) liver, kidney, and small intestine at 72 hours post-infection as determined by quantitative RT-PCR (6h n = 4; 12h n = 8; 18h n = 8; 24h n = 12; 72h n = 4). Data are shown as GE per mL of serum or GE per copy of 18S RNA for tissues. The dotted line represents the limit of detection of the assay for each tissue. (D) Levels of TNF in the serum of DENV-infected mice at 72 hours post-infection (n = 7 per group). Error bars represent the SEM and asterisks denote statistically significant differences (*, p<0.05; **, p<0.005; ***, p<0.0005; ns = not significant). Results are representative of two independent experiments.

To evaluate how STAT1 and STAT2 deficiency impacts the control of DENV infection, wild type, STAT1^−/−^, STAT2^−/−^, STAT1^−/−^/2^−/−^, and STAT1^−/−^/AR^−/−^ mice were infected intravenously with 10^10^ GE of S221, and viral RNA levels in various tissues were measured via qRT-PCR at 6, 12, 18, 24, and 72 hours after infection. Although minimal viral RNA was detected in each strain at 6 hours post-infection, increasing viremia was observed in all strains except wild type by 12 hours post-infection. At 12, 18, and 24 hours post-infection, the high viremia observed in the single- and double-deficient strains (all p<0.0005) demonstrates that the combined function of both STAT1 and STAT2 is required for effective control of viral replication **(** **Figure 2A** **)**. Previous studies have shown that the spleen is an initial site of DENV replication in mice [28], [34], and at 18 and 24 hours post-infection, levels of DENV RNA in the spleen of each knockout mouse strain were significantly higher than wild type (14- to 27-fold increase; all p<0.0005) **(** **Figure 2B** **)**. Although each single-deficient mouse strain possessed similar levels of viral RNA as STAT1^−/−^/2^−/−^ mice at 12 and 18 hours post-infection, the levels of virus in the serum and spleen were 6–7 fold higher in STAT1^−/−^ mice than STAT2^−/−^ mice by 24 hours. This suggests that early control of DENV replication requires the combined function of STAT1 and STAT2, but STAT2-independent mechanism(s) begin to restrict replication by 24 hours post-infection. At 72 hours post-infection, viremia in both STAT1^−/−^ and STAT2^−/−^ mice was reduced relative to the 24-hour time point (STAT1^−/−^ 30-fold, p<0.0001; STAT2^−/−^ 33-fold, p = 0.0019), and the levels of viremia were similar between these two strains. In contrast to the single-deficient mice, viremia in STAT1^−/−^/2^−/−^ mice increased 25-fold between 24 and 72 hours after infection. By 72 hours, viral RNA levels in the spleen had decreased significantly in all mouse strains, but remained at least 40-fold higher in STAT1^−/−^/2^−/−^ mice as compared to the single-deficient strains.

In the liver, kidney, and small intestine, the later sites of viral replication in DENV-infected mice [28], [34], significantly higher (100 to 10,000-fold) levels of viral RNA were observed in the double-deficient mice at 72 hours post-infection as compared with the single-deficient animals **(** **Figure 2C** **)**. Viral burden in STAT1^−/−^/AR^−/−^ mice was equivalent to STAT1^−/−^/2^−/−^ mice in all tissues at 72 hours post-infection (data not shown). In wild type mice, minimal or no DENV RNA was detected in these organs. In the single-deficient mice, viral RNA levels in STAT1^−/−^ and STAT2^−/−^ animals were similar in the liver, kidney, and small intestine, although viral load in the kidney was slightly higher in STAT2^−/−^ mice (4-fold; p = 0.0105).

Collectively, these data demonstrate that efficient control of early viral replication requires the combined action of STAT1 and STAT2. However, each can function independently of the other to limit viral replication later during infection. Only the combined absence of both STAT1 and STAT2 ablates the antiviral response, and results in unrestricted viral replication.

Our previous studies have shown that viral load is directly linked to high levels of circulating tumor necrosis factor (TNF), which contributes to the early death phenotype observed in S221-infected AG129 mice [27], [35]. To compare STAT1^−/−^/2^−/−^ mice to single-deficient and wild type mice, serum TNF levels were measured by ELISA. Both STAT1^−/−^ and STAT2^−/−^ mice possessed similar levels of TNF in the serum at 72 hours post-infection, which were 4–5 fold higher than that detected in wild type mice **(** **Figure 2D** **)**. However, levels of serum TNF in the single-deficient strains remained significantly lower (>20-fold) than in STAT1^−/−^/2^−/−^ mice, indicating that control of viral replication by STAT1 or STAT2 alone results in reduced TNF expression during DENV infection, and is consistent with the survival studies where only double-deficient animals were susceptible to DENV-induced death.

### Type I IFN expression in response to DENV infection is delayed in STAT-deficient mice

**Figure 3:**
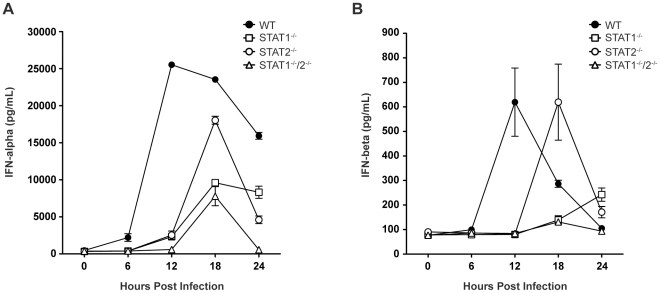
Levels of type I IFN in the serum of DENV-infected mice. Mice were infected i.v. with 10^10^ GE of S221, and serum collected at 6, 12, 18, or 24 hours post-infection. Levels of (A) IFN-α and (B) IFN-β in the serum were determined by ELISA (n = 4–6 mice per group). Error bars represent the SEM. Results are representative of two independent experiments.

Based on the survival data indicating that STAT1-independent protection is mediated via type I IFN signaling, we next examined type I IFN levels in infected mice, as expression of type I IFN is partially dependent upon a positive feedback mechanism [2]. Specifically, type I IFN levels in wild type and single-deficient mice were compared to STAT1^−/−^/2^−/−^ mice, which are not expected to possess the positive feedback loop. Serum levels of IFN-α and IFN-β in wild type, STAT1^−/−^, STAT2^−/−^, and STAT1^−/−^/2^−/−^ mice were measured by ELISA between 0 and 24 hours post-infection. High levels of serum IFN-α were present in wild type mice by 12 hours post-infection, and these levels diminished between 18 and 24 hours after infection **(** **Figure 3A** **)**, consistent with our previous observations in wild type C57BL/6 mice following DENV infection [28]. However, robust IFN-α production in both STAT1^−/−^ and STAT2^−/−^ mice was delayed until 18 hours following infection, and STAT1^−/−^ mice exhibited decreased expression of IFN-α compared with STAT2^−/−^ mice. Serum IFN-α levels in STAT1^−/−^/2^−/−^ mice followed the same kinetics as STAT1^−/−^ mice, including increased expression at 18 hours post-infection. However, despite similar levels of viremia detected in both strains at 24 hours post-infection **(** **Figure 2A** **)**, IFN-α in STAT1^−/−^ mice remained elevated but in STAT1^−/−^/2^−/−^ mice it decreased to the level at or below the limit of detection (p<0.0001). Similar trends were observed for IFN-β production following DENV infection **(** **Figure 3B** **)**, including delayed induction in the knockout strains relative to wild type mice, and elevated IFN-β levels in STAT1^−/−^ but not STAT1^−/−^/2^−/−^ mice at 24 hours. Together, these results demonstrate that the combined function of STAT1 and STAT2 is required for maximum early induction of type I IFN in response to DENV infection. The presence of either STAT1 or STAT2 alone is also sufficient to drive type I IFN expression, but with delayed kinetics when compared to an intact IFN signaling pathway. Additionally, the difference in IFN levels between STAT1^−/−^ and the double knockout mice at 24 hours shows that a STAT2-dependent mechanism contributes to type I IFN expression in the absence of STAT1.

### STAT2 induces ISG transcription independently of STAT1 during DENV infection

**Figure 4:**
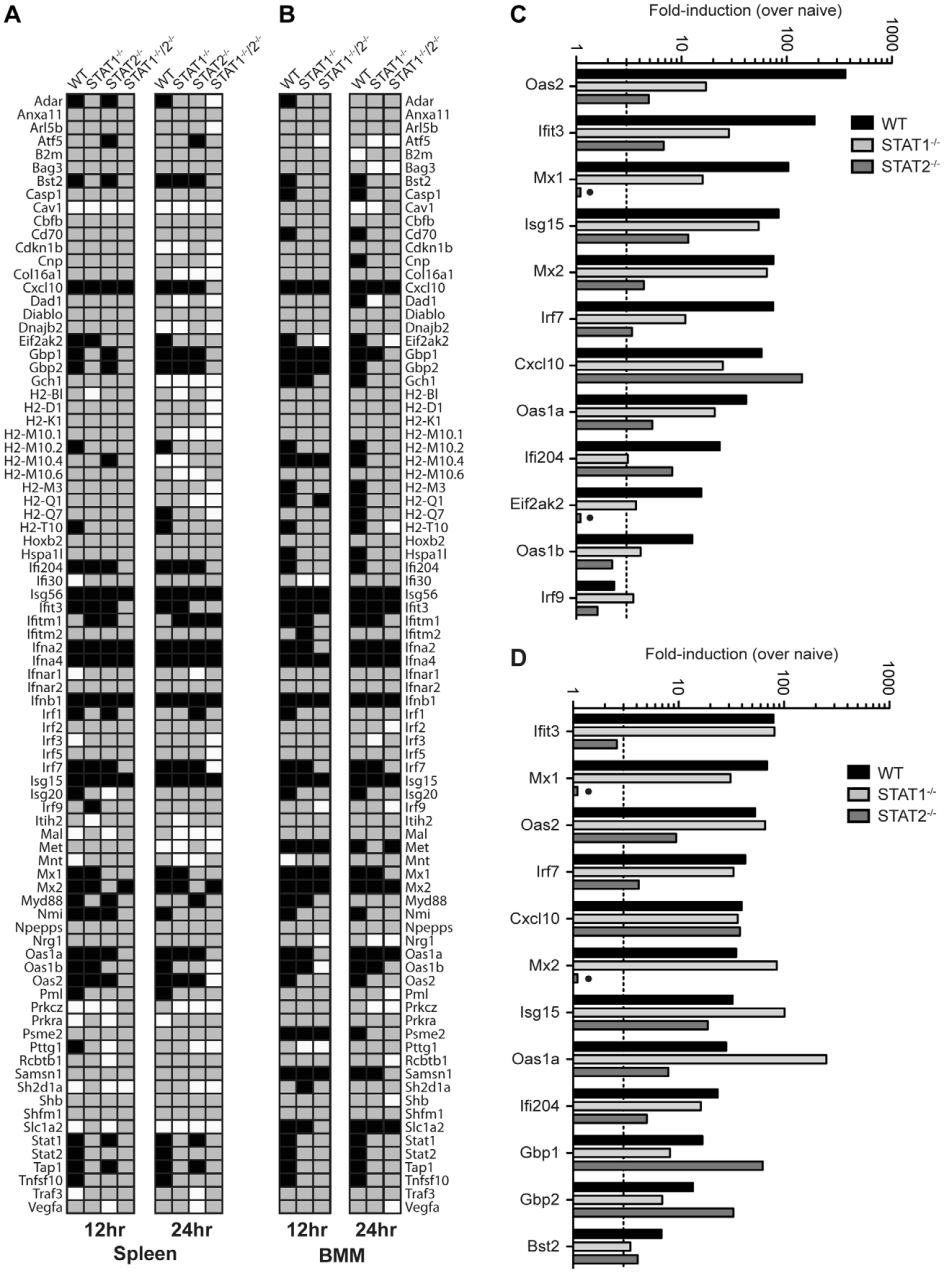
Comparison of ISG Induction in DENV-infected mice. Gene induction evaluated using the RT^2^ Profiler PCR Array (Mouse IFN-α/β Response Array; SABioscience). (A,B) Annotated gene expression of transcripts with a difference in expression of 3-fold or more over naïve (upregulation-black; downregulation-white) at 12 and 24 hours post-infection of (A) spleens isolated from wild type, STAT1^−/−^, STAT2^−/−^, and STAT1^−/−^/2^−/−^ mice infected with S221, and (B) BMMs isolated from wild type, STAT1^−/−^, and STAT1^−/−^/2^−/−^ mice infected with S221 (MOI = 5). (C, D) Gene induction in the spleen expressed as fold-increase over naïve for each strain at (C) 12 hours and (D) 24 hours post-infection. Data represent mean fold-induction of three animals per strain at each timepoint.

To further define the mechanisms by which type I IFN receptor-STAT2 signaling protects against DENV infection *in vivo*, we next examined gene expression in the spleens of S221-infected mice using the Type I IFN-related RT^2^ Profiler PCR Array (SABiosciences/QIAGEN). RNA from whole spleens was analyzed and fold-induction calculated by comparing infected mice to naïve mice of the corresponding strain. Of the 84 genes in the array, 31 (36.9%), 16 (19.0%), 22 (26.2%), and 7 (8.3%) genes, respectively, were induced 3-fold over naïve in wild type, STAT1^−/−^, STAT2^−/−^, and STAT1^−/−^/2^−/−^ mice at 12 hours post-infection **(** **Figure 4A** **)**, and the number of genes upregulated was similar at 24 hours post-infection for each mouse strain. Genes that were induced in STAT1^−/−^/2^−/−^ mice identified ISGs that were upregulated independently of either STAT1 or STAT2. These ISGs included *Ifna2*, *Ifna4*, and *Ifnb1*, which were induced to high levels in all four strains at 12 hours (>1000-fold) and 24 hours (>100-fold) post-infection **(Table S1)**, and likely represent early IRF3-mediated gene expression [36].

A number of ISGs were identified that were induced >3-fold over uninfected controls in STAT1^−/−^ mice, but were not induced in STAT1^−/−^/2^−/−^ mice **(** **Figure 4A** **)**, revealing that a STAT2-dependent mechanism can drive the expression of multiple ISGs in the absence of STAT1. In addition, several genes that were weakly upregulated in STAT1^−/−^/2^−/−^ mice (*Cxcl10*, *Mx2*, *Isg15*) were induced 6- to 28-fold higher in STAT1^−/−^ mice than in the double knockout, suggesting that the STAT1-independent pathway heavily contributes to the regulation of these genes as well. Genes upregulated in STAT1^−/−^ mice were then compared to wild type and STAT2^−/−^ mice at 12 hours (12 genes) **(** **Figure 4C** **)** and 24 hours (12 genes) **(** **Figure 4D** **)** post-infection. At the 12 hour time point, more than half of the genes in this group displayed at least 3-fold higher induction in wild type mice than in STAT1^−/−^ mice. However, by 24 hours post-infection, the gene induction levels observed in wild type and STAT1^−/−^ mice were similar (<2-fold) except for the highly elevated *Oas1a* gene in STAT1^−/−^ mice. This increased upregulation in STAT1^−/−^ mice at 24 hours is consistent with the elevated levels of type I IFN observed at 24 hours post-infection **(** **Figure 3** **)**. Two genes upregulated in STAT1^−/−^ mice, *Mx1* and *Eif2ak2 (PKR)*, were not induced in STAT2^−/−^ mice, suggesting that STAT2 is required for their expression in response to DENV infection. Conversely, *Gbp1* and *Gbp2* were induced more efficiently in STAT2^−/−^ animals than in STAT1^−/−^ mice, and several additional genes in this array were induced in STAT2^−/−^ and wild type but not STAT1^−/−^ mice at both timepoints. **(Table S1)**. Collectively, these results demonstrate that a STAT1-independent pathway regulates the expression of ISGs in a STAT2-dependent manner during DENV infection *in vivo*.

In order to perform more mechanistic studies, bone marrow-derived macrophages (BMMs) were isolated from wild type and knockout mice, and evaluated *in vitro*. BMMs were chosen because cells of the monocyte/macrophage lineage are presumed to be one of the major cell types that support DENV replication in humans and mice [37]–[39]. BMMs were infected with S221 (MOI = 5), and gene induction at 12 and 24 hours post-infection was evaluated using the RT^2^ Profiler PCR Array (**Table S2**). A majority of genes induced in the spleen were also upregulated in BMMs from each strain (29/31 genes in wild type; 13/16 in STAT1^−/−^; 5/7 in STAT1^−/−^/2^−/−^), indicating that gene regulation of ISGs is similar between the spleen and BMMs following DENV infection **(** **Figure 4B** **)**, and validating the use of BMMs as a suitable *in vitro* model for these studies.

### STAT2 does not require STAT1 for type I IFN-mediated activation in mouse bone marrow-derived macrophages

**Figure 5:**
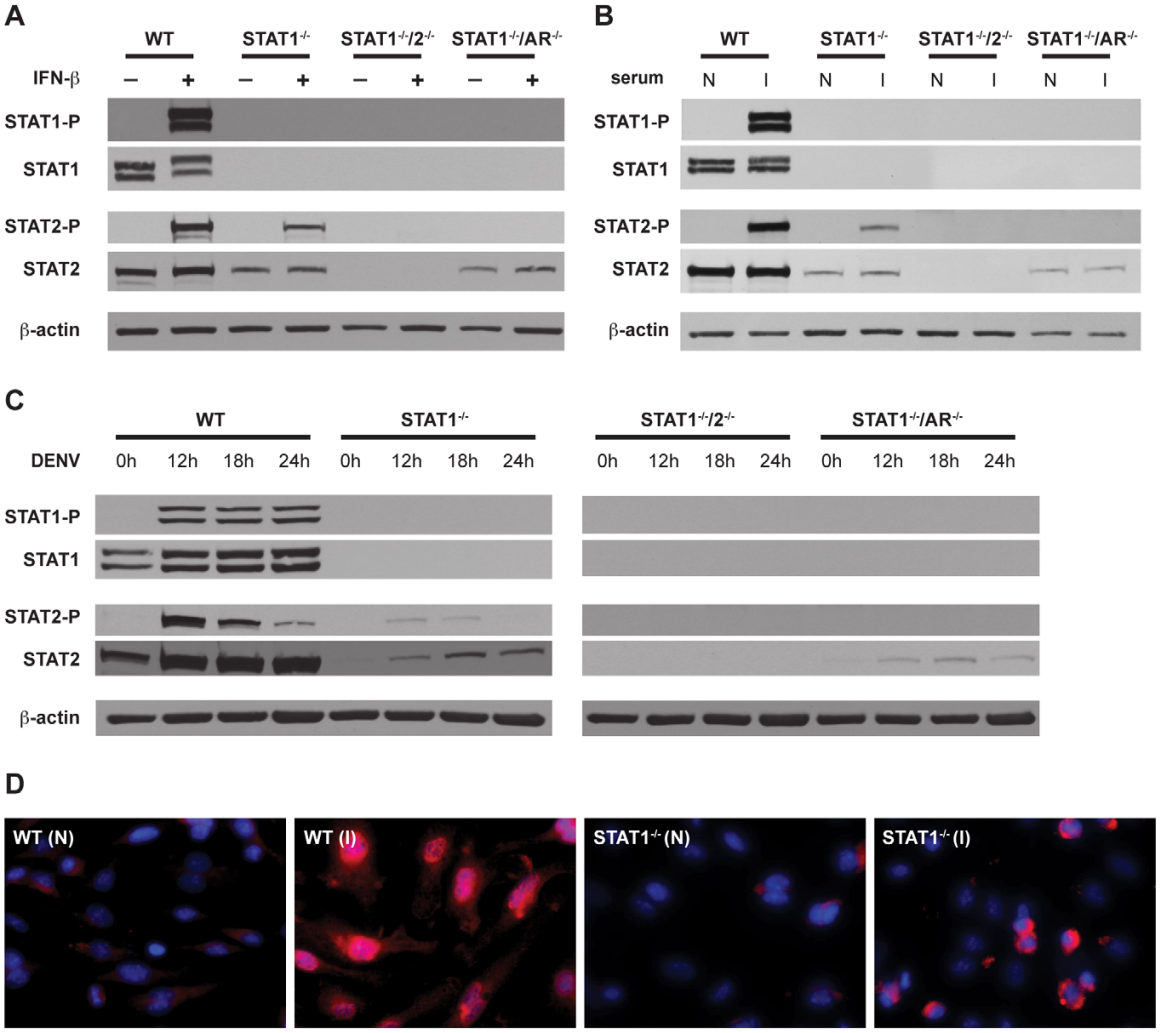
Activation of STAT1 and STAT2 in bone marrow-derived macrophages. (A–C) Whole cell lysates were generated from bone marrow-derived macrophages that were isolated from WT, STAT1^−/−^, STAT1^−/−^/2^−/−^, or STAT1^−/−^/AR^−/−^ mice and treated with (A) 1000U/mL recombinant murine IFN-β for 15 min, (B) serum from naïve (N) or S221-infected (I) mice for 1 hour, or (C) infected with S221 (MOI = 5) for the times indicated. Protein levels of STAT1, phosphorylated STAT1, STAT2, phosphorylated STAT2, and beta-actin were examined by Western blot. (D) Nuclear localization of STAT2 in bone marrow-derived macrophages isolated from WT and STAT1^−/−^ mice, and treated with serum from naïve (N) or S221-infected (I) mice for 1 hour. STAT2 (red), and DAPI (blue). Original magnification, ×400. Images are representative of each strain. Data are representative of two or more individual experiments.

To confirm activation of STAT2 in the absence of STAT1, phosphorylation and nuclear localization of STAT2 was examined in BMMs. Type I IFN signaling activates STAT2 via phosphorylation of a tyrosine residue (Y689), which is required for its association with STAT1 and incorporation into the transcriptionally active complex ISGF3 [40]. BMMs isolated from wild type, STAT1^−/−^, STAT1^−/−^/2^−/−^, and STAT1^−/−^/AR^−/−^ mice were stimulated with recombinant IFN-β for 15 minutes, and the phosphorylation status of STAT2 was examined via Western blot **(** **Figure 5A** **)**. As expected, both STAT1 and STAT2 were phosphorylated in wild type cells. Phosphorylation of STAT2 was observed in STAT1^−/−^ but not STAT1^−/−^/AR^−/−^ cells, although the basal level of STAT2 was lower in both STAT1^−/−^ and STAT1^−/−^/AR^−/−^ cells than in wild type cells. These results show that STAT2 activation can occur in the absence of STAT1 following type I IFN stimulation.

### STAT2 activation occurs independently of STAT1 following DENV infection of mouse bone marrow-derived macrophages

In addition to type I IFN, cytokines produced during DENV infection are able to activate JAK-STAT signaling, including IL-2, IL-4, IL-6, IL-10, and IFN-γ [35], [41], [42]. To examine STAT2 activation in the context of DENV infection, BMMs were incubated with serum taken from wild type mice infected with S221, and analyzed by Western blot **(** **Figure 5B** **)**. Similar to recombinant IFN-β, the infected mouse serum induced both STAT1 and STAT2 phosphorylation in wild type cells. Phosphorylation of STAT2 was detected in STAT1^−/−^ but not STAT1^−/−^/AR^−/−^ BMMs, indicating that activation of STAT2 in these cells is primarily dependent upon type I IFN signaling.

To verify that phosphorylation of STAT2 occurs in the absence of STAT1 during DENV infection, BMMs were infected with S221 and STAT phosphorylation was examined at 12, 18, and 24 hours post-infection **(** **Figure 5C** **)**. Consistent levels of STAT1 phosphorylation were detected at all time points in wild type cells, whereas levels of phosphorylated STAT2 were highest at 12 hours after infection and decreased over time. Phosphorylated STAT2 was also detected at the 12 hour and 18 hour time points in STAT1^−/−^ cells, but not in STAT1^−/−^/AR^−/−^ cells.

To further confirm STAT1-independent activation of STAT2, wild type and STAT1^−/−^ BMMs were incubated with serum from either naïve or S221-infected wild type mice for 1 hour, and nuclear localization of STAT2 was examined using immunofluorescence **(** **Figure 5D** **)**. Although the staining pattern within the nucleus appeared different between the two strains, STAT2 was observed in the nuclei of both wild type and STAT1^−/−^ cells. Taken together, these results indicate that STAT2 is activated via type I IFN receptor signaling during DENV infection, even in the absence of STAT1.

### STAT2 directly associates with ISG promoters following DENV infection

**Figure 6:**
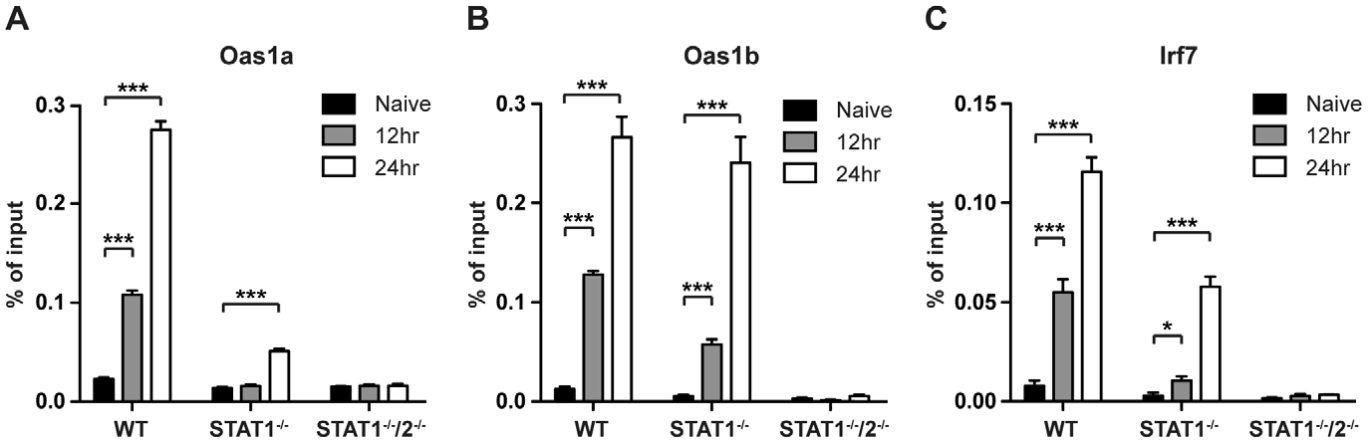
Association of STAT2 protein with ISG promoters. ChIP analysis using a STAT2-specific antibody in bone marrow-derived macrophages isolated from WT, STAT1^−/−^, or STAT1^−/−^/2^−/−^ mice and infected with S221 (MOI = 5) for 12 and 24 hours. Enrichment was measured by quantitative PCR, and percent of input DNA was determined by comparing cycle threshold value (C_t_) obtained with immunoprecipitated DNA and C_t_ value obtained from input DNA. Genes analyzed were (A) *Oas1a* (B) *Oas1b* and (C) *Irf7*. Error bars represent the SEM, and asterisks denote statistically significant differences (*, p<0.05; ***, p<0.0005). Results are representative of two independent pull-down experiments.

In response to type I IFN signaling, the STAT1:STAT2:IRF9 (ISGF3) complex translocates to the nucleus to bind ISREs found in the promoters of many ISGs. To determine whether STAT2 is directly involved in the transcriptional regulation of ISGs in the absence of STAT1, chromatin immunoprecipitation (ChIP) experiments were performed using a STAT2-specific antibody. Gene targets *Oas1a*, *Oas1b*, and *IRF7* were chosen from ISGs that were expressed in wild type and STAT1^−/−^ BMMs, but not in STAT1^−/−^/2^−/−^ double deficient cells as determined by the PCR array analysis **(Table S2)**. DNA from S221-infected wild type, STAT1^−/−^ and STAT1^−/−^/2^−/−^ BMMs was harvested at 12 and 24 hours post-infection, and quantitative PCR was performed on anti-STAT2 precipitated DNA using primer pairs specific to ISREs located in the promoters of these genes. Following DENV infection, increased binding of STAT2 to all three gene promoters was observed in wild type and STAT1^−/−^ BMMs when compared to naïve cells **(** **Figure 6A–C** **)**. STAT2 binding to the *Oas1a* promoter in STAT1^−/−^ cells was only significantly enriched at 24 hours post-infection, while *Oas1b* and *IRF7* had significant enrichment at both time points. In particular, the binding activity of STAT2 to the *Oas1b* promoter in STAT1^−/−^ cells almost matched that of wild type cells **(** **Figure 6B** **)**. Nearly undetectable DNA enrichment was observed for all three genes in STAT1^−/−^/2^−/−^ cells, providing evidence of the antibody's specificity for STAT2. Taken together, these results demonstrate that STAT2 binds to the promoters of ISGs in the absence of STAT1, providing further evidence of a direct role for STAT2 in the regulation of antiviral responses.

## Discussion

STAT1 was originally described as a transcription factor essential for type I and II IFN signaling, and initial studies described STAT1^−/−^ cells and mice as unresponsive to both type I and II IFN [8], [9]. However, later studies in STAT1^−/−^ mice infected with Sindbis virus or MCMV revealed the existence of a STAT1-independent antiviral mechanism [20]. Similarly, we have demonstrated that a STAT1-independent IFN response confers protection from DENV infection in mice [24]. Given that both type I and type II IFN-mediated responses restrict DENV infection *in vitro* and *in vivo* [25], [26], [43], [44], we were interested in defining the mechanism of this STAT1-independent protection.

In this study, we demonstrated that the combined loss of both STAT1 and STAT2 in mice resulted in early death following DENV infection. These results recapitulated the early death phenotype of AG129 mice and implied that the increased DENV susceptibility in STAT1^−/−^/2^−/−^ mice was due to the loss of type I and II IFN signaling. Conversely, both STAT1 and STAT2 single-deficient mice survived infection with DENV at this same dose of virus, suggesting there are sufficient compensatory mechanisms that protect against DENV infection in the absence of either one of these STAT proteins. While the susceptibility of the STAT1^−/−^/IFNAR^−/−^ double knockout mice to DENV infection indicated that STAT2 was functioning downstream of type I IFN signaling, the survival of STAT1^−/−^/IFNGR^−/−^ mice confirmed the potency of STAT2-mediated protection. IFNAR^−/−^/IFNGR^−/−^ (AG129) mice do not survive infection, yet STAT1^−/−^/IFNGR^−/−^ mice remain healthy, demonstrating that STAT2 alone provides sufficient signaling via the type I IFN pathway for a functional antiviral response, despite the absence of both STAT1 and type II IFN signaling. However, STAT1^−/−^ mice challenged with a 100-fold higher dose of virus succumbed to DENV infection, indicating that STAT2-mediated protection can be overcome. In contrast, STAT2^−/−^ mice remained healthy at the highest dose tested, most likely due to an intact type II IFN signaling pathway, and indicating that functional STAT1 plays a larger role in anti-DENV responses than STAT2. This claim is supported by the viral load data, where tissue viral burden in STAT2^−/−^ mice remained slightly lower than their STAT1^−/−^ counterparts at multiple timepoints following infection. Although little difference was observed in tissue viral load between the single deficient STAT mice and the double knockouts during the first 24 hours following infection, both STAT1 and STAT2 were able to function independently of each other to provide significant protection in all tissues by 72 hours post infection. Consistent with the kinetics of type I IFN expression, the viral load data suggest that antiviral responses in the single deficient mice are delayed compared to wild type mice. This delayed response is likely due to the inability to form ISGF3 (STAT1:STAT2:IRF9), the primary transcription complex associated with type I IFN signaling, resulting in suboptimal activation of the type I IFN signaling pathway. Despite the inability to form ISGF3, STAT1^−/−^ mice still induced a subset of ISGs in response to DENV infection that were not upregulated in STAT1^−/−^/2^−/−^ mice. The PCR array data, although far less comprehensive than genome-wide analysis, provided evidence that the STAT1-independent pathway mediates protection through the expression of antiviral genes.

One concern with the PCR array approach in whole spleens is that total RNA was isolated from a heterogeneous cell population, and differences in cell type distribution between strains or naïve versus infected mice could complicate analysis. To address this, PCR arrays were performed using BMMs from the different knockout strains. Although the kinetics and efficiency of infection are likely to be different *in vivo* versus *in vitro*, the overall pattern of ISG induction in BMMs was similar to the spleen, confirming that our gene expression data were not biased by using whole tissue. Furthermore, flow cytometry analysis of both naïve and infected mouse spleens revealed that little difference exists in cellular composition between STAT1^−/−^ and STAT1^−/−^/2^−/−^ mice **(Figure S1)**, whose relative differences in gene induction were compared to identify STAT1-independent ISGs.

Prior to this work, several key studies demonstrated that STAT2 can function in the absence of STAT1. Hahm and colleagues showed that dendritic cell maturation is impaired in a IFN-β-dependent, STAT2-dependent manner following infection of pluripotent bone marrow cells by measles virus (MV) and LCMV [31]. Although this was the first description of a STAT2-mediated STAT1-independent phenotype, the focus of this work was on an immunosuppressive rather than an antiviral phenotype. Sarkis and colleagues first demonstrated that STAT2 was required for IFN-α-induced expression of several antiviral genes in cultured liver cells, including *Mx1*, *PKR*, and *Isg15* [45], independently of STAT1 expression. However, the authors inferred this STAT2-dependent pathway was liver-specific since they could not carry these observations into cells other than human hepatoma cells. Similarly, Lou and colleagues also described STAT1-independent gene induction of RIG-G by STAT2 in response to type I IFN, in an *in vitro* system that required the over-expression of IRF9 [46]. The present study extends these bodies of work by demonstrating that STAT2 can mediate antiviral responses independently from STAT1 through direct association with ISG promoters, including antiviral genes that are otherwise regulated by ISGF3, not just ISGs that have been demonstrated to be STAT2-dependent. To our knowledge, this is the first report of STAT2 functioning downstream of type I IFN signaling in the absence of STAT1, which results in a functional antiviral response *in vivo*.

ChIP analysis confirmed the association of STAT2 protein with antiviral gene promoters, implying that STAT2 has a direct role in transcriptional regulation, but the exact mechanism of how STAT2 mediates IFN signaling remains to be determined. Although it possesses a strong transactivation domain, STAT2 binds DNA poorly, suggesting that STAT2 does not function alone [47]. IRF9 (p48) provides both DNA binding and sequence specificity functions to the ISGF3 complex [47], and its involvement has been implicated in STAT1-independent mechanisms [45], [46]. Therefore, the most likely candidate for an alternative STAT2 complex includes IRF9. In addition to ISGF3, several complexes containing STAT2 have been described, including STAT2:3 and STAT2:6 heterodimers, and STAT2:2 homodimers [47]–[49]. However, further biochemical studies will be required to identify the components of this alternative transcriptional complex that functions in the absence of STAT1. The promoter sequence element that binds this alternative complex also remains unknown, and likely depends upon other binding partners. The ISGs identified by PCR array in this study represented a subset of ISGs induced in wild type mice, suggesting a novel binding element is unlikely. A more comprehensive study of gene modulation other than the PCR array, such as full microarray or ChIP-seq, should shed light on how ISRE, GAS, and other promoter elements are regulated in the absence of STAT1.

It is important to consider the difference between mouse and human STAT2 when interpreting the results of our studies. While the other STAT proteins have a high degree of sequence homology between the two species, the C-terminal transactivation domains between human and mouse STAT2 are completely divergent, so it is unknown whether mouse and human STAT2 function similarly when taken out of the context of ISGF3. However, *in vitro* studies have suggested mouse STAT2 is functionally analogous to human STAT2 in ISGF3 formation, ISRE activation, and biological response to type I IFN [50]. The relevance of species-specific STAT2 has recently been demonstrated in terms of DENV infection: the NS5 protein of DENV binds and targets human STAT2 for proteasomal degradation to inhibit type I IFN responses [16], [18]. Ashour and colleagues have recently demonstrated that this targeting is species specific, and murine STAT2 is resistant to NS5-mediated degradation [17]. In the studies presented here, basal expression of STAT2 was unaffected in BMMs infected with DENV, but the level of infection of our BMMs may be as low as 1% (unpublished observation), suggesting NS5 has little direct effect upon STAT2 in our culture system.

In this study, we have identified STAT2 as a key component of the STAT1-independent mechanism of protection against DENV infection in mice, and demonstrated that both STAT1 and STAT2 possess the ability to independently limit the severity of DENV pathogenesis. For many viruses, inhibition of STAT-mediated signaling is a major mechanism to evade antiviral responses. Our data suggests that DENV-mediated inactivation of STAT1 function alone is not sufficient to neutralize antiviral responses, emphasizing the importance of DENV mechanisms to specifically target host STAT2 function. Increasing evidence suggests that the relative ability of flaviviruses to subvert STAT signaling, including DENV, WNV, JEV, and Kunjin viruses, may be a contributing factor to their virulence [51]–[54]. In conjunction, epidemiological studies have revealed that selection for virulent DENV strains occurs in both humans and mosquitoes [55]–[57]. A more detailed understanding of how DENV causes specific pathologies, even in an immunodeficient setting (as in STAT1^−/−^/2^−/−^ mice), may offer additional insight regarding prevention of human DENV-induced disease from both current and newly emerging strains.

## Materials and Methods

### Ethics statement

This study was carried out in strict accordance with the recommendations in the Guide for the Care and Use of Laboratory Animals of the National Institutes of Health, the US Public Health Service Policy on Humane Care and Use of Laboratory Animals, and the Association for Assessment and Accreditation of Laboratory Animal Care International (AAALAC). All experimental procedures were pre-approved and performed according to the guidelines set by the La Jolla Institute for Allergy and Immunology Animal Care and Use Committee.

### Cells and viruses

Generation and preparation of DENV2 strain S221 has been described previously [28], [35]. Briefly, S221 is a triple-plaque-purified clone of D2S10, which was generated by alternative passage between AG129 mice and C6/36 mosquito cells. Viral stocks used for these studies were grown in C6/36 mosquito cells and concentrated by ultracentrifugation [34]. Genome equivalents (GE) were quantified by real-time reverse transcription-PCR (RT-PCR) and infectious titer determined by plaque assay on BHK-21 cells, as previously described [34].

### Mouse experiments

Wild type (WT) 129/Sv/Ev were purchased from Taconic Farms. STAT1^−/−^ 129/Sv/Ev mice bearing a deletion in the DNA binding domain of the *STAT1* gene were obtained from Dr. Joan Durbin (Ohio State University, Columbus, OH). STAT2^−/−^ 129/Sv/Ev mice were obtained from Dr. Christian Schindler (Columbia University, New York, NY). All mice were bred and housed under specific pathogen-free conditions at the La Jolla Institute for Allergy and Immunology (LIAI). For all experiments, sex-matched mice at 5 to 6 weeks of age were infected intravenously with 10^10^ GE (≈2×10^5^ PFU) of S221 diluted into 200µL PBS with 5% FCS. For survival studies, mice were sacrificed when moribund or at the first signs of paralysis.

### Viral RNA harvest and quantification

Mice were euthanized via isoflurane inhalation and blood was collected by cardiac puncture. Mice were perfused with 60mL phosphate-buffered saline (PBS) and tissues collected into RNALater (QIAGEN). Tissues were homogenized for 3 minutes at 4°C in 1mL tissue lysis buffer (QIAGEN RLT Buffer) using a Mini-beadbeater-8 (BioSpec Products). Liver and small intestine homogenates were diluted 5-fold and homogenized for 3 additional minutes. RNA was isolated from tissue homogenates using the RNeasy minikit (QIAGEN) and from 30µL serum using the QIAmp viral RNA minikit (QIAGEN) according to manufacturer's instructions, eluted and stored at −80°C until analysis. Quantitative RT-PCR to detect DENV and 18S was performed as previously described [34]. Viral load is expressed as GE per mL in serum, or GE normalized to copies of 18S in tissues.

### ELISA

Serum from infected or naïve animals was analyzed using either a mouse TNF-α ELISA Ready-Set-Go kit (eBioscience) or mouse IFN-α ELISA and IFN-β ELISA (PBL Technologies), all according to the manufacturers' instructions. The IFN-α ELISA kit detects all subtypes of IFN-α according to the manufacturer.

### Isolation and DENV infection of bone marrow-derived macrophages

To generate bone marrow-derived macrophages, bone marrow from femur and tibia of 5- to 7-week old mice were isolated and cultured for 6–7 days in RPMI 1640 (10% fetal calf serum, penicillin, streptomycin, HEPES, sodium pyruvate, 55µM β-mercaptoethanol) in the presence of 100ng/mL murine macrophage stimulating factor (M-CSF) (PeproTech). On day 6–7 of culture, cells were treated with either 1000U recombinant murine IFN-β (PBL Biomed) for 15 minutes, 100µL serum obtained from DENV2-infected wild type mice for 60 minutes, or infected with S221 (MOI = 5), followed by harvesting of the cells at 0, 12, 18, or 24 hours post-infection.

### Western blot analysis

Immediately following treatment, bone marrow-derived macrophages (10^6^ cells) were lysed in Western lysis buffer (25mM Tris-HCl, 150mM NaCl, 1% NP40) in the presence of protease inhibitor cocktail (Sigma), Phosphatase Inhibitor Cocktail Set II (Calbiochem), and 1mM okadaic acid (Sigma). Samples were resolved on 8% SDS-polyacrylamide gels, transferred overnight to PVDF (Millipore) at 4°C, and probed overnight with the following antibodies: anti-STAT1 and anti-P701-STAT1 (Cell Signaling), anti-STAT2 (Millipore), anti-P689-STAT2 (Santa Cruz Biotech), and anti-beta-actin (Sigma). Blots were incubated with HRP-conjugated secondary antibody (Pierce) and visualized with Western Lightning Plus-ECL (Perkin Elmer).

### PCR Arrays

Splenic RNA was harvested from spleens as described above. Bone marrow-derived macrophage RNA was harvested using Trizol reagent (Invitrogen) and further purified with the RNeasy minikit (QIAGEN). To remove genomic DNA contaminants, 1µg total RNA was treated with 0.5 units DNAse (Invitrogen) for 15 minutes at 20°C and then 8 minutes at 70°C to inactivate DNAse enzyme. cDNA was then generated using the RT^2^ First Strand kit (SABioscience) according to the manufacturer's protocol. Newly synthesized cDNA was loaded onto RT^2^ Profiler (SABioscience) 384-well PCR array plates (PAMM-016) and amplified on the LightCycler 480 PCR system (Roche) for 40 cycles. The resulting threshold cycle values (C_t_) were uploaded onto the SABioscience website (http://www.sabiosciences.com/pcr/arrayanalysis.php) and relative levels of gene expression (fold differences) were calculated using the provided software. C_t_ values ≥35 cycles were considered undetectable.

### Immunofluorescence

Bone marrow-derived macrophages were seeded on glass coverslips and treated with infected mouse serum for 1 hour. Following treatment, cells were fixed in 3% paraformaldehyde for 30 minutes, permeabilized in 0.1% Triton X-100 for 10 minutes, and blocked in 20% purified goat serum (Pierce) for 1 hour. Cells were washed, incubated with anti-STAT2 antibody (obtained from Dr. Christian Schindler) followed by DyLight 649-labeled goat anti-rabbit IgG (Jackson Immunoresearch). Nuclei were counterstained with DAPI, and viewed using a Marianas deconvolution fluorescence microscope (3i).

### Chromatin immunoprecipitation (ChIP)

ChIP assays were performed essentially as described [58]. Bone marrow-derived macrophages (3×10^6^ cells) were fixed in 1% formaldehyde and lysed in 50mM Tris-HCl, pH 8.0, 10mM EDTA, 1% SDS on ice for 5 minutes. Lysates were sonicated and diluted with 9 parts 50mM Tris-HCl, pH 8.0, 167mM NaCl, 1.1% Triton X-100, and 0.11% sodium deoxycholate. 10µg of DNA was used for each immunoprecipitation. Rabbit polyclonal antibody specific for murine STAT2 (Schindler) was used to precipitate STAT2-DNA complexes. Immunocomplexes were bound to protein G-agarose beads (Pierce Thermo Scientific), washed 5 times, and eluted by incubating beads at 65°C overnight in 10mM Tris-HCl, 5mM EDTA, 300mM NaCl, 0.5% SDS. Eluted DNA was treated with RNAse A and proteinase K, and purified using the QIAquick PCR Purification kit (QIAGEN). Quantitative SYBR Green PCR was performed on the LightCycler 480 PCR System (Roche) for 45 cycles. Primer sequences used: Oas1a(+) AAACCCCAAGAAAGCCAGAT, Oas1a(−) CTCCCAGCCTAGCTGAAATG; Oas1b(+) CTGTTCAGAAGCCCTAACGC; Oas1b(−) AGGTCAGCACAGAAGCTGGT; IRF7(+) TGGGATCTGAGTAAGGGTCG; IRF7(−) GCCAAGGTGGCTGTAGATGT.

### Flow cytometry

Mice were infected i.v. with 10^10^ GE of S221. Single-cell suspensions were prepared from naïve and infected spleens harvested at 12 and 24 hours post-infection with Spleen Dissociation Medium (Stem Cell Technologies) (n = 3 per group). The following antibodies were used: Alexa Fluor 647-conjugated anti-B220 (HI30; Biolegend) and anti-F4/80 (Cl:A3-1; Biolegend); eFluor 450-conjugated anti-CD4 (RM4-5; eBioscience) and anti-Gr-1 (RB6-8C5; eBioscience); fluorescein isothiocyanate-conjugated anti-CD3ε (145-2C11; eBioscience) and anti-CD11b (M1/70; BD Pharmingen); phycoerythrin-conjugated anti-CD11c (N418; eBioscience); phycoerythrin-indotricarbocyanine-conjugated anti-CD8α (53-6.7; BD Pharmingen); and peridinin chlorophyll protein-cyanine 5.5-conjugated anti-B220 (RA3-6B2; BD Pharmingen) and anti-CD49b (DX5; Biolegend). Samples were collected on FACS LSR II (BD Biosciences) and were analyzed with FlowJo software (TreeStar). Live cells were counted with a Vi-CELL XR (Beckman Coulter).

### Statistical analysis

For all studies, data were analyzed by Prism 5 software (GraphPad Software). For survival studies, Kaplan-Meier survival curves were analyzed by log rank test. Viral load, cytokine ELISA, and ChIP data were analyzed using the unpaired *t* test.

## Supporting Information

Figure S1 Cell composition of spleens following DENV infection. Percentage of various live cell types in splenocytes isolated from mice infected with 10^10^ GE of S221 at 0 hours (naïve), 12 hours and 24 hours post-infection. Each were classified: (A) macrophages-CD11b^+^F4/80^+^, (B) CD4^+^ T cells-CD3^+^CD4+, (C) CD8^+^ T cells-CD3^+^CD8^+^, (D) natural killer cells-CD3^−^CD49b^+^, (E) NK T cells-CD3^+^CD49b^+^, (F) B cells-B220^+^, (G) neutrophils-CD11b^+^Gr-1^+^, (H) plasmacytoid dendritic cells-CD11c^+^B220^+^, and other dendritic cells-CD11c^+^B220^−^. (I) Total live cell counts for each strain ± SEM. Error bars represent the SEM and asterisks denote statistically significant differences (*, p<0.05; **, p<0.005). Results are mean values from three animals.
(5.04 MB TIF)

Table S1 Quantitative PCR Array data from infected spleens. Complete list of genes examined in the IFN-α/β related PCR array (PAMM-016; SABiosciences). Fold change was calculated based upon naïve control mice for each individual strain using software provided by the manufacturer (see Material and Methods).
(5.41 MB TIF)

Table S2 Quantitative PCR Array data from infected bone marrow derived macrophages. Complete list of genes examined in the IFN-α/β related PCR array (PAMM-016; SABiosciences). Fold change was calculated based upon naïve control mice for each individual strain using software provided by the manufacturer (see Material and Methods).
(5.31 MB TIF)
