# Supplementary figures and images for: STAT2 Mediates Innate Immunity to Dengue Virus in the Absence of STAT1 via the Type I Interferon Receptor
Source: PLoS Pathog. 2011 Feb 17;7(2):e1001297. doi: 10.1371/journal.ppat.1001297 (PMC3040673; doi:10.1371/journal.ppat.1001297)

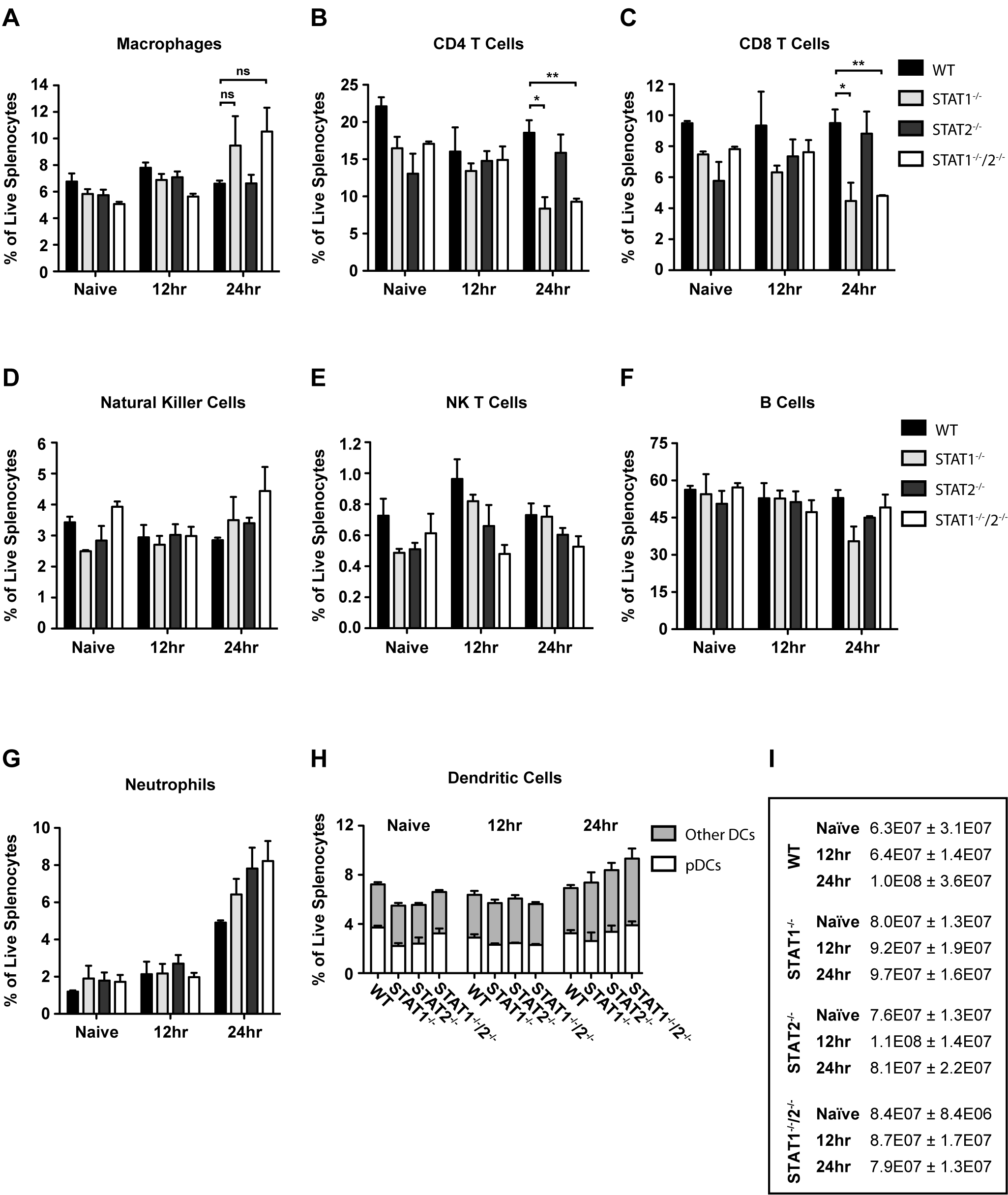

Supplement: Figure S1 — Cell composition of spleens following DENV infection. Percentage of various live cell types in splenocytes isolated from mice infected with 1010 GE of S221 at 0 hours (naïve), 12 hours and 24 hours post-infection. Each were classified: (A) macrophages-CD11b+F4/80+, (B) CD4+ T cells-CD3+CD4+, (C) CD8+ T cells-CD3+CD8+, (D) natural killer cells-CD3−CD49b+, (E) NK T cells-CD3+CD49b+, (F) B cells-B220+, (G) neutrophils-CD11b+Gr-1+, (H) plasmacytoid dendritic cells-CD11c+B220+, and other dendritic cells-CD11c+B220−. (I) Total live cell counts for each strain ± SEM. Error bars represent the SEM and asterisks denote statistically significant differences (*, p<0.05; **, p<0.005). Results are mean values from three animals. (5.04 MB TIF) [file ppat.1001297.s001.tif]

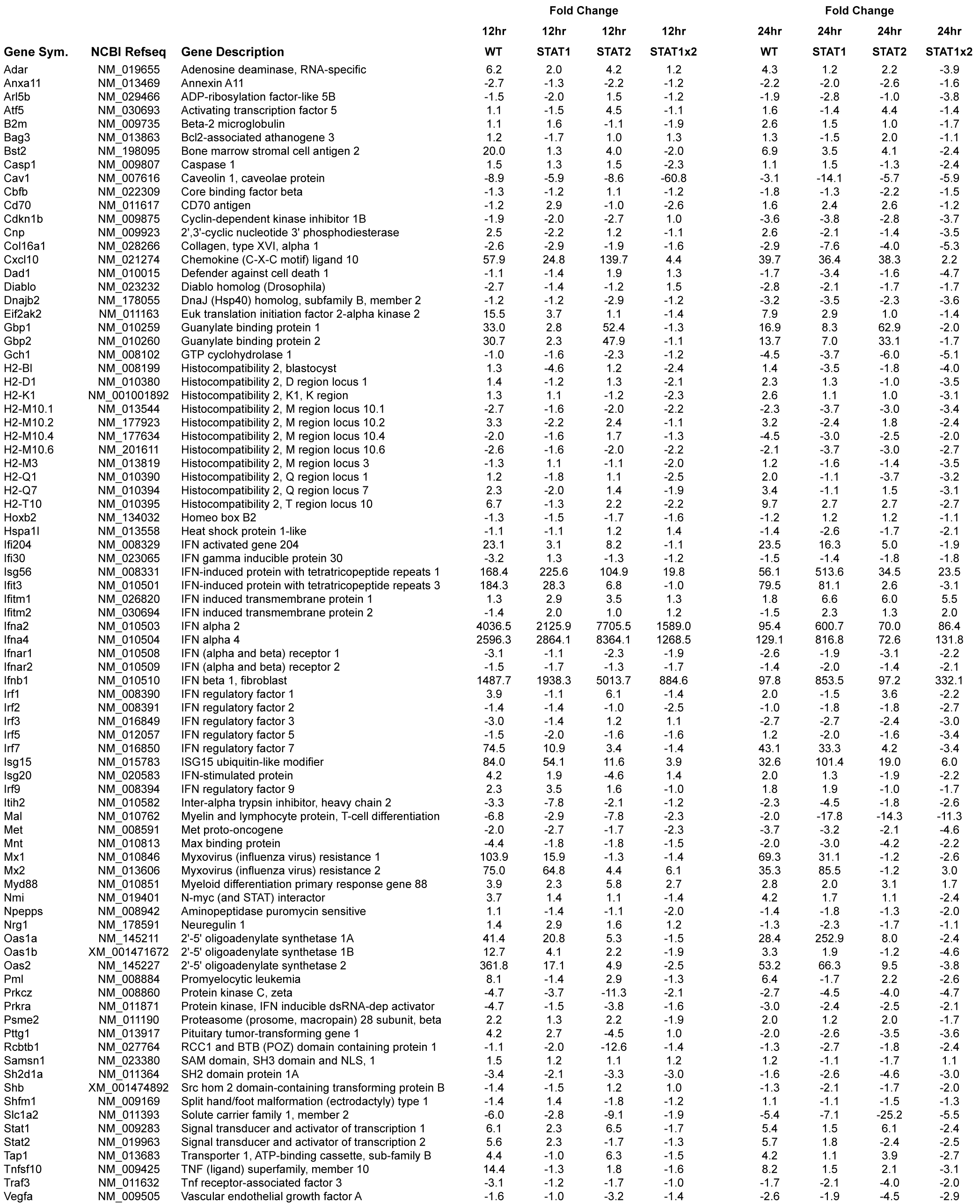

Supplement: Table S1 — Quantitative PCR Array data from infected spleens. Complete list of genes examined in the IFN-α/β related PCR array (PAMM-016; SABiosciences). Fold change was calculated based upon naïve control mice for each individual strain using software provided by the manufacturer (see Material and Methods). (5.41 MB TIF) [file ppat.1001297.s002.tif]

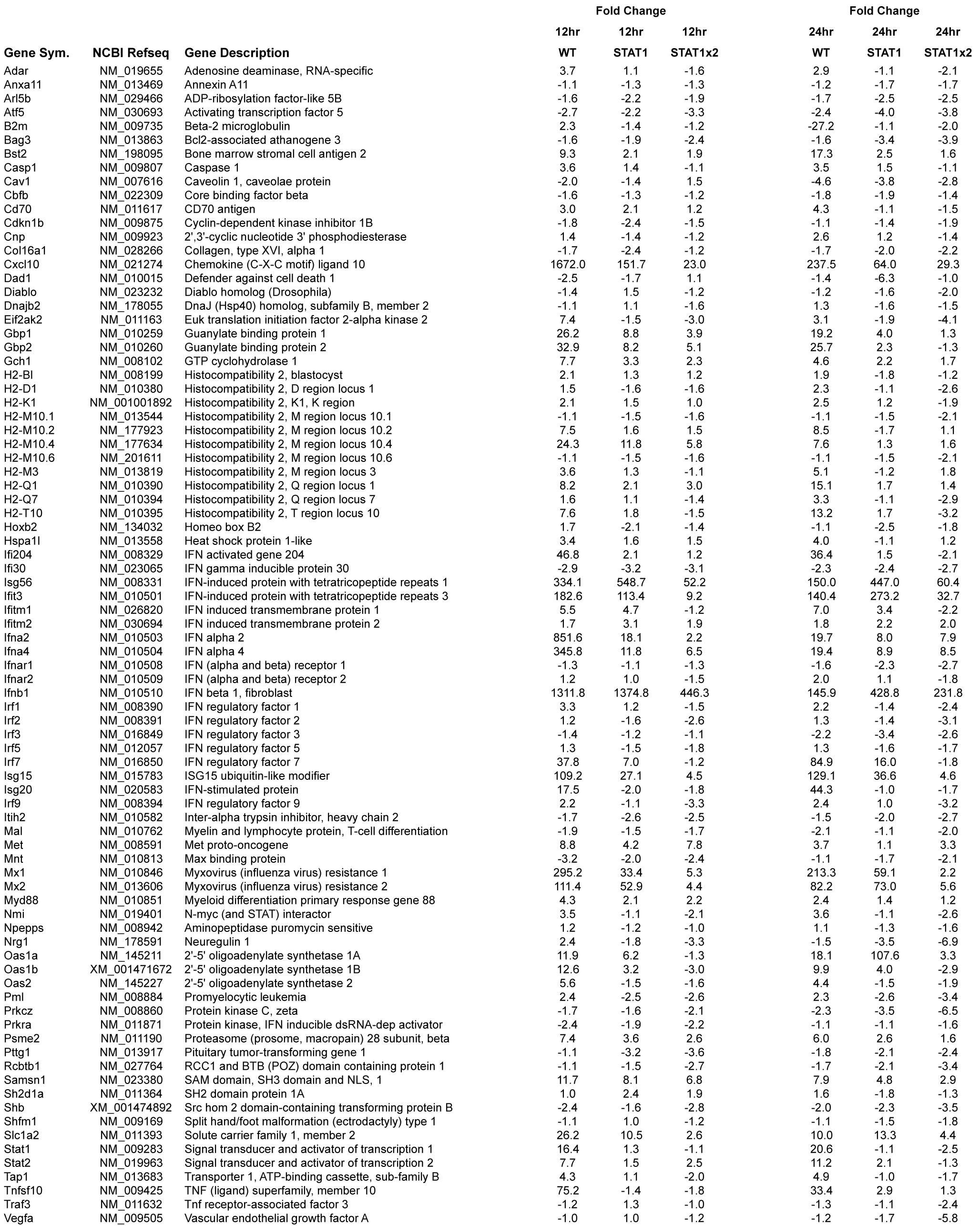

Supplement: Table S2 — Quantitative PCR Array data from infected bone marrow derived macrophages. Complete list of genes examined in the IFN-α/β related PCR array (PAMM-016; SABiosciences). Fold change was calculated based upon naïve control mice for each individual strain using software provided by the manufacturer (see Material and Methods). (5.31 MB TIF) [file ppat.1001297.s003.tif]
